# *N*,*N*′-Dicyclo­hexyl­naphthalene-1,8;4:5-dicarboximide

**DOI:** 10.1107/S1600536808025221

**Published:** 2008-08-09

**Authors:** Deepak Shukla, Manju Rajeswaran

**Affiliations:** a Eastman Kodak Company, Kodak Research Laboratories, Rochester, NY 14650-2106, USA

## Abstract

The title compound, C_26_H_26_N_2_O_4_, synthesized by the reaction of naphthalene-1,4,5,8-tetra­carboxylic acid anhydride and cyclo­hexyl­amine, exhibits good *n*-type semiconducting properties. Accordingly, thin-film transistor devices comprising this compound show *n*-type behavior with high field-effect electron moblity *ca* 6 cm^2^/Vs [Shukla, Nelson, Freeman, Rajeswaran, Ahearn, Meyer & Carey(2008 ▶). *Chem. Mater.* Submitted]. The asymmetric unit comprises one-quarter of the centrosymmetric mol­ecule in which all but two methyl­ene C atoms of the cyclo­hexane ring lie on a mirror plane; the point-group symmetry is 2/*m*. The naphthalene­diimide unit is strictly planar, and the cyclo­hexane rings adopt chair conformations with the diimide unit in an equatorial position on each ring.

## Related literature

For general background on the semi-conducting properties and use of this class of material in organic thin-film transistor applications, see: Chesterfield *et al.* (2004*a* ▶,*b* ▶); Facceti *et al.* (2008 ▶); Jones *et al.* (2004 ▶); Katz *et al.* (2000*a* ▶,*b* ▶); Shukla *et al.* (2008 ▶).
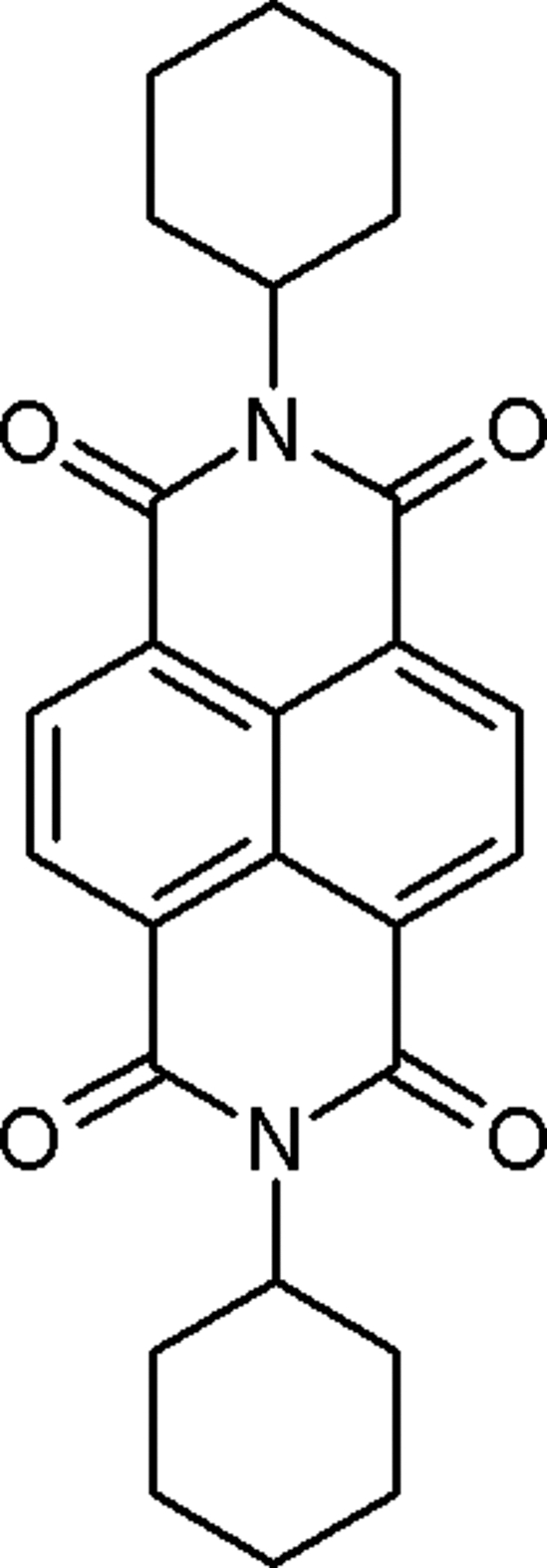


## Experimental

### 

#### Crystal data

C_26_H_26_N_2_O_4_
*M* *_r_* = 430.49
Monoclinic, 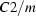

*a* = 8.5410 (2) Å
*b* = 6.6780 (2) Å
*c* = 18.4270 (9) Å
β = 102.4790 (18)°
*V* = 1026.19 (6) Å^3^
*Z* = 2
Mo *K*α radiation
μ = 0.09 mm^−1^
*T* = 293 (2) K
0.35 × 0.25 × 0.17 mm

#### Data collection

Nonius KappaCCD diffractometer
Absorption correction: none
3354 measured reflections
1227 independent reflections
787 reflections with *I* > 2σ(*I*)
*R*_int_ = 0.087

#### Refinement

*R*[*F* ^2^ > 2σ(*F* ^2^)] = 0.067
*wR*(*F* ^2^) = 0.182
*S* = 1.06
1227 reflections
91 parameters
H-atom parameters constrained
Δρ_max_ = 0.39 e Å^−3^
Δρ_min_ = −0.29 e Å^−3^

### 

Data collection: *COLLECT* (Nonius, 2000 ▶); cell refinement: *SCALEPACK* (Otwinowski & Minor, 1997 ▶); data reduction: *DENZO* (Otwinowski & Minor, 1997 ▶) and *SCALEPACK*; program(s) used to solve structure: *SIR97* (Altomare *et al.*, 1999 ▶); program(s) used to refine structure: *SHELXTL* (Sheldrick, 2008 ▶); molecular graphics: *SHELXTL*; software used to prepare material for publication: *publCIF* (Westrip, 2007 ▶).

## Supplementary Material

Crystal structure: contains datablocks I, global. DOI: 10.1107/S1600536808025221/sj2528sup1.cif

Structure factors: contains datablocks I. DOI: 10.1107/S1600536808025221/sj2528Isup2.hkl

Additional supplementary materials:  crystallographic information; 3D view; checkCIF report

## Figures and Tables

**Fig. 1.**
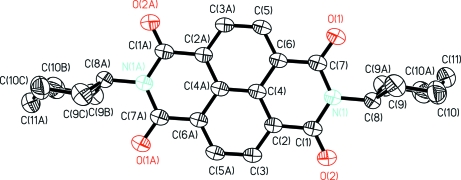
Structure of the title compound (I), with the atom numbering scheme. Displacement ellipsoids are drawn at the 50% probability level and H atoms are omitted for clarity.

## supplementary crystallographic information

### Comment

Amongst n-type semiconductors, naphthalene diimide (NDI) and perylene diimide (PDI) based systems have been studied extensively (Chesterfield, *et al.*, 2004*a*; Chesterfield *et al.*, 2004*b*; Facceti *et al.*,  2008; Jones, *et al.*, 2004; Katz, *et al.*, 2000*a*; Katz, *et al.*, 2000*b*). We report here the structure of the title diimide molecule, I, (Fig. 1).

### Experimental

The diimide 1 was prepared by direct condensation of 1,4,5,8-naphthalenetetracarboxylic acid anhydride (1.34 g, 5.00 mmol) and cyclohexylamine (30 mmol) in the presence of zinc acetate (50 mg) in 15 mL quinoline. The mixture was heated at 140-150°C for four hours, cooled and diluted with several volumes of methanol. The resulting slurry was filtered, the collected solid washed with methanol and dried in air. The crude product was then purified by train sublimation at 10^-4^ to 10^-6^ torr. ^1^H NMR (CD_2_Cl_2_,500.05 MHz): δ (ppm) = 8.76 (s, 4H), 5.10 (t,2H, J = 12 Hz), 2.64 (dt, 2H, J = 12 and 11.7 Hzs), 1.57 (dt, 2H, J = 12 and11.7 Hz), 2.03 (d, 2H, J = 12 Hz), 1.87 (d, 2H, J = 12 Hz), 1.47 (m, 2H); ^13^C(CD_2_Cl_2_, 500.05 MHz): d = 163.23, 130.74, 127.13, 126.70,54.85, 29.38, 26.66, 25.52; MS (MALDI-TOF) *m/z* cald. for [C_26_H_26_N_2_O_4_]430.5 found: 430.2.

### Refinement

All H-atoms were positioned geometrically using a riding model with d(C-H) = 0.93Å,  U_iso_=1.2U_eq_ (C) for aromatic 0.97Å,  U_iso_ = 1.2U_eq_ (C) for CH_2_ atoms.

### Crystal data

**Table:**

C_26_H_26_N_2_O_4_	*F*_000_ = 456
*M**_r_* = 430.49	*D*_x_ = 1.393 Mg m^−^^3^
Monoclinic, *C*2/*m*	Mo *K*α radiation λ = 0.71073 Å
Hall symbol: -C 2y	Cell parameters from 21067 reflections
*a* = 8.5410 (2) Å	θ = 1.0–27.5º
*b* = 6.6780 (2) Å	µ = 0.09 mm^−^^1^
*c* = 18.4270 (9) Å	*T* = 293 (2) K
β = 102.4790 (18)º	Block, orange
*V* = 1026.19 (6) Å^3^	0.35 × 0.25 × 0.17 mm
*Z* = 2	

### Data collection

**Table:**

Nonius KappaCCD diffractometer	1227 independent reflections
Radiation source: fine-focus sealed tube	787 reflections with *I* > 2σ(*I*)
Monochromator: graphite	*R*_int_ = 0.087
Detector resolution: 9 pixels mm^-1^	θ_max_ = 27.4º
*T* = 293(2) K	θ_min_ = 4.3º
φ and ω scans	*h* = −10→10
Absorption correction: none	*k* = −8→8
3354 measured reflections	*l* = −23→20

### Refinement

**Table:**

Refinement on *F*^2^	Secondary atom site location: difference Fourier map
Least-squares matrix: full	Hydrogen site location: inferred from neighbouring sites
*R*[*F*^2^ > 2σ(*F*^2^)] = 0.067	H-atom parameters constrained
*wR*(*F*^2^) = 0.182	*w* = 1/[σ^2^(*F*_o_^2^) + (0.0638*P*)^2^ + 1.0546*P*] where *P* = (*F*_o_^2^ + 2*F*_c_^2^)/3
*S* = 1.06	(Δ/σ)_max_ < 0.001
1227 reflections	Δρ_max_ = 0.39 e Å^−^^3^
91 parameters	Δρ_min_ = −0.29 e Å^−^^3^
Primary atom site location: structure-invariant direct methods	Extinction correction: none

### Special details

**Table:**

Geometry. All e.s.d.'s (except the e.s.d. in the dihedral angle between two l.s. planes) are estimated using the full covariance matrix. The cell e.s.d.'s are taken into account individually in the estimation of e.s.d.'s in distances, angles and torsion angles; correlations between e.s.d.'s in cell parameters are only used when they are defined by crystal symmetry. An approximate (isotropic) treatment of cell e.s.d.'s is used for estimating e.s.d.'s involving l.s. planes.
Refinement. Refinement of *F*^2^ against ALL reflections. The weighted *R*-factor *wR* and goodness of fit *S* are based on *F*^2^, conventional *R*-factors *R* are based on *F*, with *F* set to zero for negative *F*^2^. The threshold expression of *F*^2^ > σ(*F*^2^) is used only for calculating *R*-factors(gt) *etc*. and is not relevant to the choice of reflections for refinement. *R*-factors based on *F*^2^ are statistically about twice as large as those based on *F*, and *R*- factors based on ALL data will be even larger.

### Fractional atomic coordinates and isotropic or equivalent isotropic displacement parameters (Å^2^)

**Table:**

	*x*	*y*	*z*	*U*_iso_*/*U*_eq_	
O1	0.2160 (3)	0.0000	0.28476 (12)	0.0594 (8)	
O2	0.7630 (3)	0.0000	0.33332 (13)	0.0647 (8)	
N1	0.4894 (3)	0.0000	0.30625 (13)	0.0431 (7)	
C1	0.6391 (4)	0.0000	0.35566 (18)	0.0457 (8)	
C2	0.6418 (3)	0.0000	0.43622 (17)	0.0422 (8)	
C3	0.7854 (4)	0.0000	0.48703 (18)	0.0510 (9)	
H3	0.8806	0.0000	0.4703	0.061*	
C4	0.4979 (3)	0.0000	0.46157 (16)	0.0384 (7)	
C5	0.2086 (4)	0.0000	0.43639 (18)	0.0494 (9)	
H5	0.1100	0.0000	0.4030	0.059*	
C6	0.3486 (3)	0.0000	0.41020 (17)	0.0410 (7)	
C7	0.3427 (4)	0.0000	0.32968 (18)	0.0446 (8)	
C8	0.4868 (4)	0.0000	0.22507 (16)	0.0460 (8)	
H8	0.5991	0.0000	0.2207	0.055*	
C9	0.4125 (3)	0.1896 (4)	0.18665 (13)	0.0591 (7)	
H9A	0.4684	0.3060	0.2110	0.071*	
H9B	0.3010	0.1986	0.1901	0.071*	
C10	0.4238 (3)	0.1862 (5)	0.10529 (14)	0.0708 (9)	
H10A	0.3699	0.3031	0.0803	0.085*	
H10B	0.5356	0.1926	0.1022	0.085*	
C11	0.3488 (5)	0.0000	0.0665 (2)	0.0664 (11)	
H11A	0.3624	0.0000	0.0156	0.080*	
H11B	0.2348	0.0000	0.0654	0.080*	

### Atomic displacement parameters (Å^2^)

**Table:**

	*U*^11^	*U*^22^	*U*^33^	*U*^12^	*U*^13^	*U*^23^
O1	0.0365 (12)	0.088 (2)	0.0494 (14)	0.000	0.0005 (9)	0.000
O2	0.0363 (12)	0.105 (2)	0.0535 (14)	0.000	0.0113 (10)	0.000
N1	0.0330 (12)	0.0522 (17)	0.0428 (14)	0.000	0.0052 (10)	0.000
C1	0.0323 (15)	0.051 (2)	0.0514 (19)	0.000	0.0047 (12)	0.000
C2	0.0322 (15)	0.0478 (19)	0.0456 (18)	0.000	0.0066 (12)	0.000
C3	0.0296 (15)	0.071 (2)	0.0513 (19)	0.000	0.0072 (12)	0.000
C4	0.0309 (14)	0.0364 (16)	0.0459 (16)	0.000	0.0040 (11)	0.000
C5	0.0292 (14)	0.065 (2)	0.0503 (19)	0.000	0.0012 (12)	0.000
C6	0.0303 (15)	0.0445 (18)	0.0460 (17)	0.000	0.0033 (12)	0.000
C7	0.0357 (15)	0.0466 (19)	0.0491 (18)	0.000	0.0038 (13)	0.000
C8	0.0379 (15)	0.059 (2)	0.0403 (17)	0.000	0.0059 (12)	0.000
C9	0.0643 (15)	0.0483 (15)	0.0601 (16)	−0.0050 (13)	0.0033 (11)	0.0031 (12)
C10	0.0716 (17)	0.080 (2)	0.0561 (16)	−0.0103 (17)	0.0045 (12)	0.0170 (15)
C11	0.056 (2)	0.094 (3)	0.047 (2)	0.000	0.0059 (16)	0.000

### Geometric parameters (Å, °)

**Table:**

O1—C7	1.212 (3)	C5—H5	0.9300
O2—C1	1.216 (4)	C6—C7	1.474 (4)
N1—C1	1.401 (4)	C8—C9^ii^	1.521 (3)
N1—C7	1.411 (4)	C8—C9	1.521 (3)
N1—C8	1.491 (4)	C8—H8	0.9800
C1—C2	1.480 (4)	C9—C10	1.523 (3)
C2—C3	1.373 (4)	C9—H9A	0.9700
C2—C4	1.406 (4)	C9—H9B	0.9700
C3—C5^i^	1.401 (4)	C10—C11	1.506 (4)
C3—H3	0.9300	C10—H10A	0.9700
C4—C4^i^	1.409 (6)	C10—H10B	0.9700
C4—C6	1.415 (4)	C11—C10^ii^	1.506 (4)
C5—C6	1.382 (4)	C11—H11A	0.9700
C5—C3^i^	1.401 (4)	C11—H11B	0.9700
			
C1—N1—C7	123.2 (3)	N1—C8—C9^ii^	112.42 (17)
C1—N1—C8	117.8 (3)	N1—C8—C9	112.42 (17)
C7—N1—C8	119.0 (2)	C9^ii^—C8—C9	112.7 (3)
O2—C1—N1	121.3 (3)	N1—C8—H8	106.2
O2—C1—C2	120.9 (3)	C9^ii^—C8—H8	106.2
N1—C1—C2	117.8 (3)	C9—C8—H8	106.2
C3—C2—C4	119.3 (3)	C8—C9—C10	109.7 (2)
C3—C2—C1	120.1 (3)	C8—C9—H9A	109.7
C4—C2—C1	120.5 (3)	C10—C9—H9A	109.7
C2—C3—C5^i^	121.3 (3)	C8—C9—H9B	109.7
C2—C3—H3	119.3	C10—C9—H9B	109.7
C5^i^—C3—H3	119.3	H9A—C9—H9B	108.2
C2—C4—C4^i^	120.0 (3)	C11—C10—C9	111.6 (3)
C2—C4—C6	120.3 (3)	C11—C10—H10A	109.3
C4^i^—C4—C6	119.7 (3)	C9—C10—H10A	109.3
C6—C5—C3^i^	120.4 (3)	C11—C10—H10B	109.3
C6—C5—H5	119.8	C9—C10—H10B	109.3
C3^i^—C5—H5	119.8	H10A—C10—H10B	108.0
C5—C6—C4	119.3 (3)	C10—C11—C10^ii^	111.3 (3)
C5—C6—C7	120.5 (3)	C10—C11—H11A	109.4
C4—C6—C7	120.2 (3)	C10^ii^—C11—H11A	109.4
O1—C7—N1	120.8 (3)	C10—C11—H11B	109.4
O1—C7—C6	121.2 (3)	C10^ii^—C11—H11B	109.4
N1—C7—C6	118.0 (2)	H11A—C11—H11B	108.0
			
C7—N1—C1—O2	180.0	C2—C4—C6—C7	0.0
C8—N1—C1—O2	0.0	C4^i^—C4—C6—C7	180.0
C7—N1—C1—C2	0.0	C1—N1—C7—O1	180.0
C8—N1—C1—C2	180.0	C8—N1—C7—O1	0.0
O2—C1—C2—C3	0.0	C1—N1—C7—C6	0.0
N1—C1—C2—C3	180.0	C8—N1—C7—C6	180.0
O2—C1—C2—C4	180.0	C5—C6—C7—O1	0.0
N1—C1—C2—C4	0.0	C4—C6—C7—O1	180.0
C4—C2—C3—C5^i^	0.000 (1)	C5—C6—C7—N1	180.0
C1—C2—C3—C5^i^	180.0	C4—C6—C7—N1	0.0
C3—C2—C4—C4^i^	0.0	C1—N1—C8—C9^ii^	115.76 (19)
C1—C2—C4—C4^i^	180.0	C7—N1—C8—C9^ii^	−64.24 (19)
C3—C2—C4—C6	180.0	C1—N1—C8—C9	−115.76 (19)
C1—C2—C4—C6	0.0	C7—N1—C8—C9	64.24 (19)
C3^i^—C5—C6—C4	0.000 (1)	N1—C8—C9—C10	176.2 (2)
C3^i^—C5—C6—C7	180.0	C9^ii^—C8—C9—C10	−55.5 (4)
C2—C4—C6—C5	180.0	C8—C9—C10—C11	55.2 (3)
C4^i^—C4—C6—C5	0.000 (1)	C9—C10—C11—C10^ii^	−56.5 (4)

Symmetry codes: (i) −*x*+1, −*y*, −*z*+1; (ii) *x*, −*y*, *z*.
